# Vestibuloplasty and its impact on the long-term survival and success of dental implants in irradiated and non-irradiated patients after head and neck tumor therapy: a retrospective study

**DOI:** 10.1007/s00784-023-05096-x

**Published:** 2023-06-17

**Authors:** Jonas Wüster, Claudia Sachse, Christian Sachse, Carsten Rendenbach, Oliver Wagendorf, Kirstin Vach, Saskia Preissner, Max Heiland, Katja Nelson, Susanne Nahles

**Affiliations:** 1 Department of Oral and Maxillofacial Surgery, Campus Virchow-Klinikum, Charité – Universitätsmedizin Berlin, Corporate Member of Freie Universität Berlin, Humboldt-Universität Zu Berlin, and Berlin Institute of Health, Campus Benjamin Franklin Hindenburgdamm 30, 12203 Berlin, Germany; 2 Institute of Medical Biometry and Statistics, Faculty of Medicine, University of Freiburg, Freiburg Im Breisgau , Baden-Württemberg Germany; 3 Department of Oral and Maxillofacial Surgery, Faculty of Medicine, University Medical Center Freiburg, Albert Ludwig University of Freiburg, Freiburg Im Breisgau, Baden-Württemberg Germany

**Keywords:** Oral cancer, Prosthetic rehabilitation, Dental implants, Irradiated patients, Dental implant success, Dental implant survival

## Abstract

**Objectives:**

This study aimed to evaluate the influence of vestibuloplasty on the clinical success and survival of dental implants in head and neck tumor patients.

**Materials and methods:**

A retrospective single-center study was conducted. All patients received surgical therapy of a tumor in the head or neck and underwent surgical therapy and, if necessary, radiotherapy/radiochemotherapy. Patients with compromised soft tissue conditions received vestibuloplasty using a split thickness skin graft and an implant-retained splint. Implant survival and success and the influence of vestibuloplasty, gender, radiotherapy, and localizations were evaluated.

**Results:**

A total of 247 dental implants in 49 patients (18 women and 31 men; mean age of 63.6 years) were evaluated. During the observation period, 6 implants were lost. The cumulative survival rate was 99.1% after 1 year and 3 years and 93.1% after 5 years for patients without vestibuloplasty, compared to a survival and success rate of 100% after 5 years in patients with vestibuloplasty. Additionally, patients with vestibuloplasty showed significantly lower peri-implant bone resorption rates after 5 years (mesial: *p* = *0.003*; distal: *p* = *0.001*).

**Conclusion:**

This study demonstrates a high cumulative survival and success rate of dental implants after 5 years in head and neck tumor patients, irrespective of irradiation. Patients with vestibuloplasty showed a significantly higher rate of implant survival and significantly lower peri-implant bone resorption after 5 years.

**Clinical relevance:**

Vestibuloplasty should always be considered and applied if required by the anatomical situations to achieve high implant survival/success rates in head and neck tumor patients.

## Introduction

Functional peri-implant soft tissue is highly relevant for longevity and aesthetical outcomes in dental implantology [1–3]. Additionally, the attached gingiva surrounding an implant and abutment provides additional protection against mechanical trauma, even in compromised patients [4, 5]. Despite the similarities, the anatomy of soft tissue around dental implants differs from that around healthy teeth and can therefore lead to complications that affect implant survival [6]. Therefore, various procedures exist for increasing the peri-implant attached keratinized gingiva, such as the apically positioned flap [7] and the pedicle lingual or buccal flap with epithelial regeneration [8, 9]. Such soft tissue grafting procedures result in favorable peri-implant health, gain of keratinized mucosa, high marginal bone levels, and improved bleeding indices [10]. Another option for enhancing peri-implant attached mucosa levels is vestibuloplasty (VP), mostly performed with free palatal gingiva grafts [11, 12]. Unfortunately, shrinkage in the vertical and horizontal direction of mucosa grafts is a common problem and often limits the long-term result of VP [1]. Split-thickness palatal mucosa grafts are often inadequate as an alternative, due of their limited size. In contrast to dental implants in healthy patients, little is known about peri-implant soft tissue management in head and neck tumor patients, despite the need for optimal soft tissue conditions and a stable denture-bearing area due to the altered anatomy after tumor resection and reconstruction in the head and neck. New insights on peri-implant tissues and their ideal management in healthy patients highlight the importance of soft tissue management in patients after ablative tumor resection—especially if osseous reconstruction of the jaw is required [13]. However, to date, there is a lack of data with long-term results or a valid number of patients [14].

In 2009, Heberer and Nelson developed a gentle and satisfactory surgical method for patients with severely compromised situations after ablative tumor therapy in the head and neck, in which the shrinkage of a split skin graft was reduced while the created denture-bearing area remained stable [5]. The described VP provides a reliable procedure in the course of preprosthetic preparations that leads to an acceptably deepened vestibulum and a decrease in muscle pull [5]. To this day, to the best of the authors’ knowledge, no sufficient data exist on long-term dental implant survival and success in head and neck tumor patients with emphasis on pre-prosthetic VP, effects on the peri-implant tissue, and influences on clinical outcome.

This study aimed to evaluate the long-term survival and success of dental implants and their clinical parameters in patients after ablative tumor surgery of the oral cavity and VP. Additionally, the influence of irradiation, the localization of the implant, gender, and age was examined.

## Methods

### Study design

This retrospective single-center study was performed at the Department of Oral and Maxillofacial Surgery, Charité—Universitätsmedizin Berlin to evaluate the impact of preprosthetic VP on the survival and success of dental implants as well as on clinical parameters in head and neck tumor patients after therapy. The study protocol was designed in accordance with the Helsinki Declaration of 1964 as revised in 2013 and approved by the ethics committee of Charité—Universitätsmedizin Berlin (EA4/064/18).

### Patients

Inclusion criteria were the surgical treatment of a tumor in the head and neck, radiation/radiochemotherapy if required, and dental implant insertion no sooner than 6 months after surgery and/or radiotherapy. Patients with considerably compromised general health conditions, such as immunocompromisation (e.g., due to autoimmune disease, HIV infection, or cortisone treatment), were not included in this study. Irradiated patients were only included in the case of absolute nicotine abstinence, while non-irradiated patients were included if they belonged to the “very light daily smoker” group with an average usage rate of 1–5 cigarettes per day [15].

### Surgical treatment

**Fig. 1:**
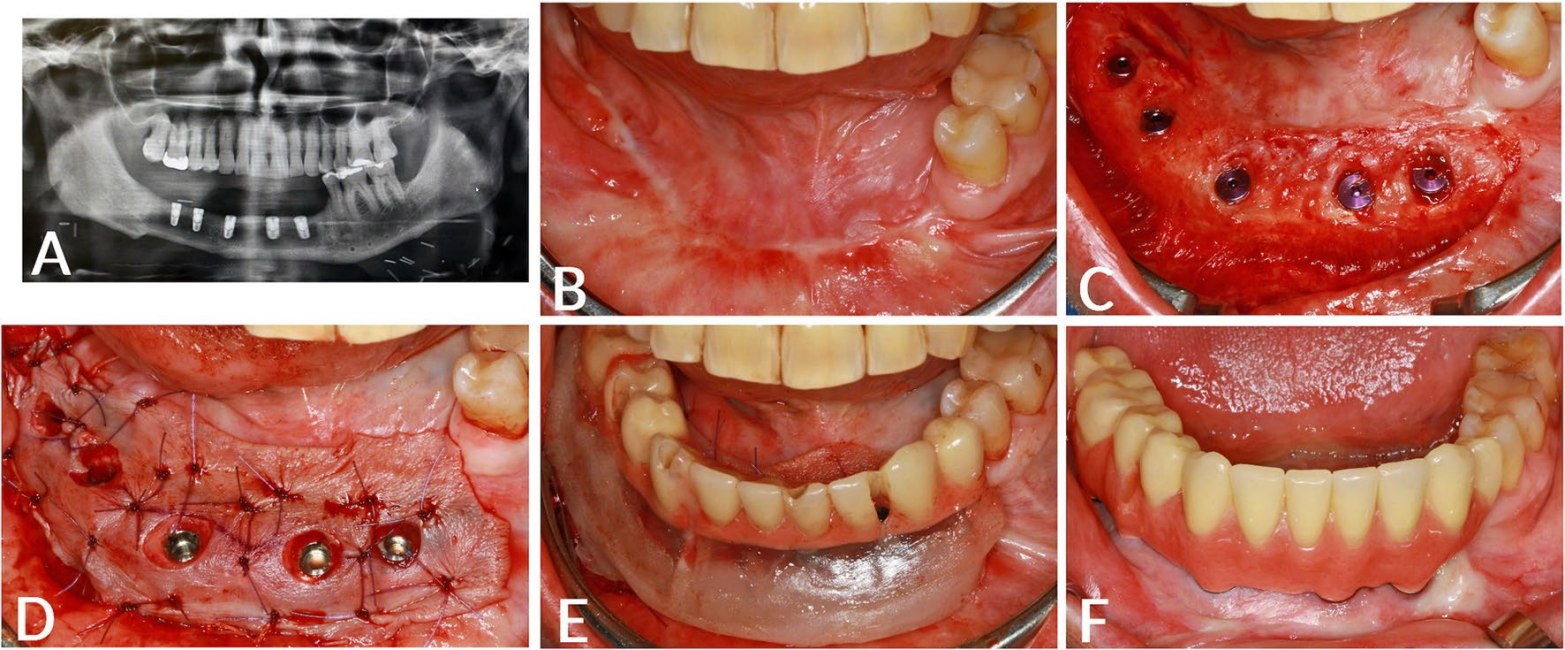
**A** Orthopantomogram after implantation of 5 dental implants in the bone of the microvascular fibula transplant. **B** Intraoral situation after implantation of 5 dental implants, before vestibuloplasty. **C** Preparation of the mucosal flap with further lingual and buccal development. **D** Application of 0.2 mm split thickness skin graft from the upper thigh and perforation above the dental implants sutured in position. **E** Relined implant supported temporary prosthetic treatment, as a healing splint according to Heberer and Nelson [5]). **F** Intraoral situation 12 months after vestibuloplasty and insertion of the intermediate prosthetic restoration

All implants were placed based on the protocol given by the manufacturer, Camlog Root-Line (Camlog Biotechnologies AG, Basel, Switzerland). All implants were placed epicrestally, under local anesthesia (Ultracain D-S forte 1:100.000). The healing time for implants was 2 months when placed in the mandible and 3 months when placed in the maxilla. No distinction was made between irradiated and non-irradiated patients. All patients received an oral antibiotic regimen of amoxicillin/clavulanic acid at a dosage of 875/125 mg every 12 hours for 1 day preoperatively and 4 days postoperatively. In patients with penicillin intolerance, clindamycin was given 3 times per day for the same period. If required, peri-implant soft tissue conditions were improved with a split skin graft according to the method described by Heberer and Nelson (Fig. 1) [5]. VP was indicated if the attached keratinized gingiva around implants was less than 1 mm in size or after microvascular reconstructive therapies had been performed on patients. Patients without microvascular reconstruction and/or attached keratinized gingiva around implants higher than 1 mm received no VP.

### Clinical evaluation

All patients underwent standardized routine clinical evaluation after implant insertion and prosthetic restoration, with a follow-up every 3 months within the first year and every 12 months subsequently [16]. During follow-up examinations, peri-implant bone level, modified bleeding index (mBI), modified plaque index (mPI) [17, 18], attached keratinized gingiva (AG), and pocket depth (PD) were measured. Mesial and distal peri-implant bone level changes were measured using the method described by Gomez-Roman et al. [19, 20]. Implant success was evaluated according to the method described by Buser et al. [21]. Mean peri-implant bone level ± standard deviation was determined by examining the mesial and distal after 1, 3, and 5 years. A subdivision was done to distinguish between VP (VP +  = VP; VP- = no VP), irradiation (R +  = irradiation; R- = non-irradiation), and irradiation with or without VP (VP + R +  = irradiation and VP; VP- R +  = irradiation without VP). Routine standardized panoramic radiographs (trademark: Planmeca ProMax; type: ProMax 3D Max, Pro Face Med Series H23 120 kV) were regularly included in follow-up controls after 1, 2, 3, and 5 years. All patients involved in this study received a dental prophylaxis by trained personnel every 3 months.

### Statistics

Relative frequencies, means, and standard deviations were calculated for a descriptive analysis of data; furthermore, boxplots and Kaplan–Meier plots were created for the graphical presentation of the data. Cox regression models with a robust estimation of standard errors and a consideration of clustering of the data were used to evaluate the influence of different covariates on survival or success. Linear mixed models were adopted to evaluate the influence of several factors on medial and distal loss and clinical tooth parameters such as PD, AG, PI, and BI. Ordered mixed logistic regression was used in case of ordinal dependent variables. In all subsequent pairwise comparisons, Scheffe’s method was applied to correct for multiple testing. Calculations were performed using the statistical software STATA 17.0 (StataCorp LT, College Station, TX, USA). The probability level for statistical significance was set at 5% (*p* = 0.05).

## Results

A total of 49 patients (18 women and 31 men) with a mean age of 63.6 years (range of 41–88 years) with 247 dental implants (89 dental implants in women and 158 in men) were enrolled in this study. Eighty-two implants were placed in the maxilla and 165 in the mandible. Within the observation period, 1 patient (with 8 dental implants; observation time of 43.5 months) died. In this case, the last clinical evaluation of the patient was included, representing the end of the observation period. All patients underwent surgical tumor resection (OSCC *n* = 39; OPSCC *n* = 7; ameloblastoma *n* = 1; odontogenic keratocyst *n* = 1; cancer of unknown primary *n* = 1); in 14 patients, the surgical procedure was followed by additional radiochemotherapy, and in 13 patients, radiotherapy was required. Radiochemotherapy was conducted over 6 weeks, with a dose of up to 50–72 Gy in fractions of 2 Gy for 5 days per week. During this time, platinum-based chemotherapy (30 mg/m^2^ of body surface area) was given once weekly in weeks 1 and 6. Subsequently, dental implant insertion was performed at least 6 months later. VP took place in 31 patients; all these patients received a VP of the mandible, and 6 patients received additional VP of the maxilla. In total, 11 patients underwent tumor resection and primary reconstruction with a fibula free flap (FFF), where 33 dental implants could be subsequently inserted in the fibula flaps. In total, 6 implants were lost in irradiated patients with no VP. For all dental implants, the cumulative survival rate was 99.6% at 1, 2, and 3 years and 97.1% at 5 years. General dental implant success was 99.6% after 1, 2, and 3 years and 97.1% after 5 years.

### Gender

All dental implant losses were recorded in men, who showed an implant survival rate of 99.4% after 3 years and 95.9% after 5 years. The dental implant success rate in women was 100% for the first 5 years, compared to 99.4% after 1 and 3 years and 95.6% after 5 years in men. No significant differences were found between the genders regarding dental implant survival.

### Jaw

The dental implant survival rate was 98.8% for the maxilla after 1, 2, 3, and 5 years; for the mandible, it was 100% after 3 years and 96.3% after 5 years (*p* = *0.54*). Dental implant success was 98.8% after 1, 2, 3, and 5 years for the maxilla, compared to 100% in the first 3 years and 96.2% after 5 years for the mandible.

### Radiation therapy

The cumulative implant survival rate in irradiated patients was 99.3% at 1, 2, and 3 years and reached 95.7% at 5 years. In 22 patients with merely a surgical tumor and no additional therapy, a total of 98 dental implants were placed. Among these 22 patients, the cumulative implant survival rate remained 100% after 1, 2, 3, and 5 years. No significant difference appeared (*p* = *0.985*). The same applies for the implant success rate, which was 100% after 1, 3, and 5 years in non-irradiated patients. The implant success rate in irradiated patients was 99.3% after 1, 3, and 5 years.

### Vestibuloplasty

**Fig. 2:**
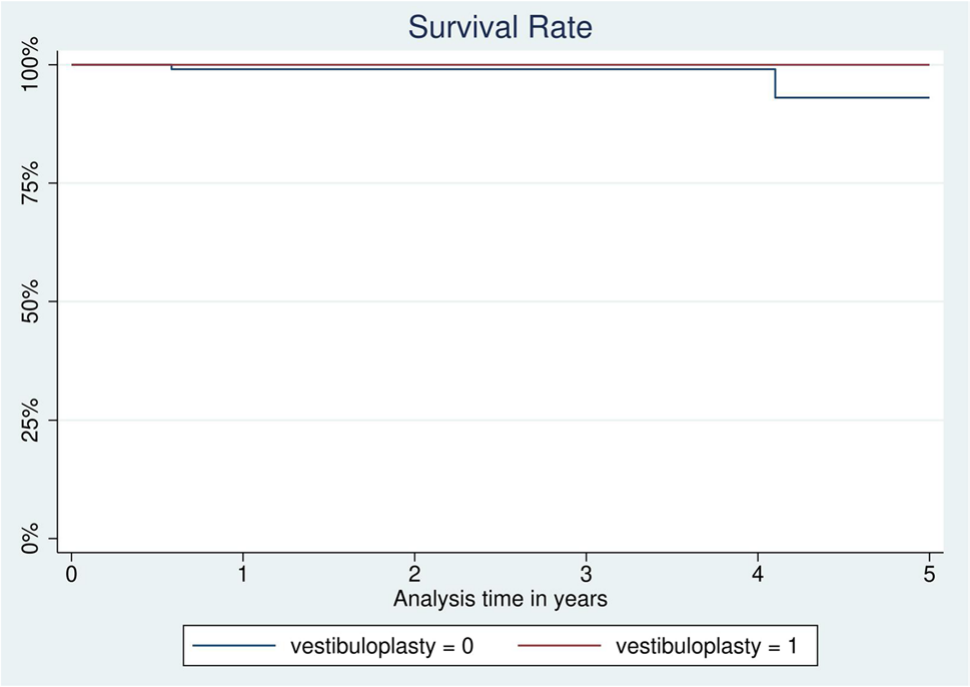
Kaplan*–*Meier survival analysis for implants with (vestibuloplasty=1) or without vestibuloplasty (vestibuloplasty=0), according to Heberer and Nelson

Patients with VP received 140 of 247 dental implants and had a cumulative implant survival rate of 100% at 1, 2, 3, and 5 years (Fig. 2). The cumulative survival rate of implants in patients without VP was 99.1% for the first 3 years and 93.1% after 5 years. The effect of VP on implant survival was statistically significant (*p* < *0.014*). Patients with VP had a dental implant success rate of 100% after 1, 3, and 5 years. Dental implants in patients without VP had a lower success rate of 99.1% after 1 and 3 years. After 5 years, the success rate decreased to 93.5%.

### Clinical parameters

**Fig. 3:**
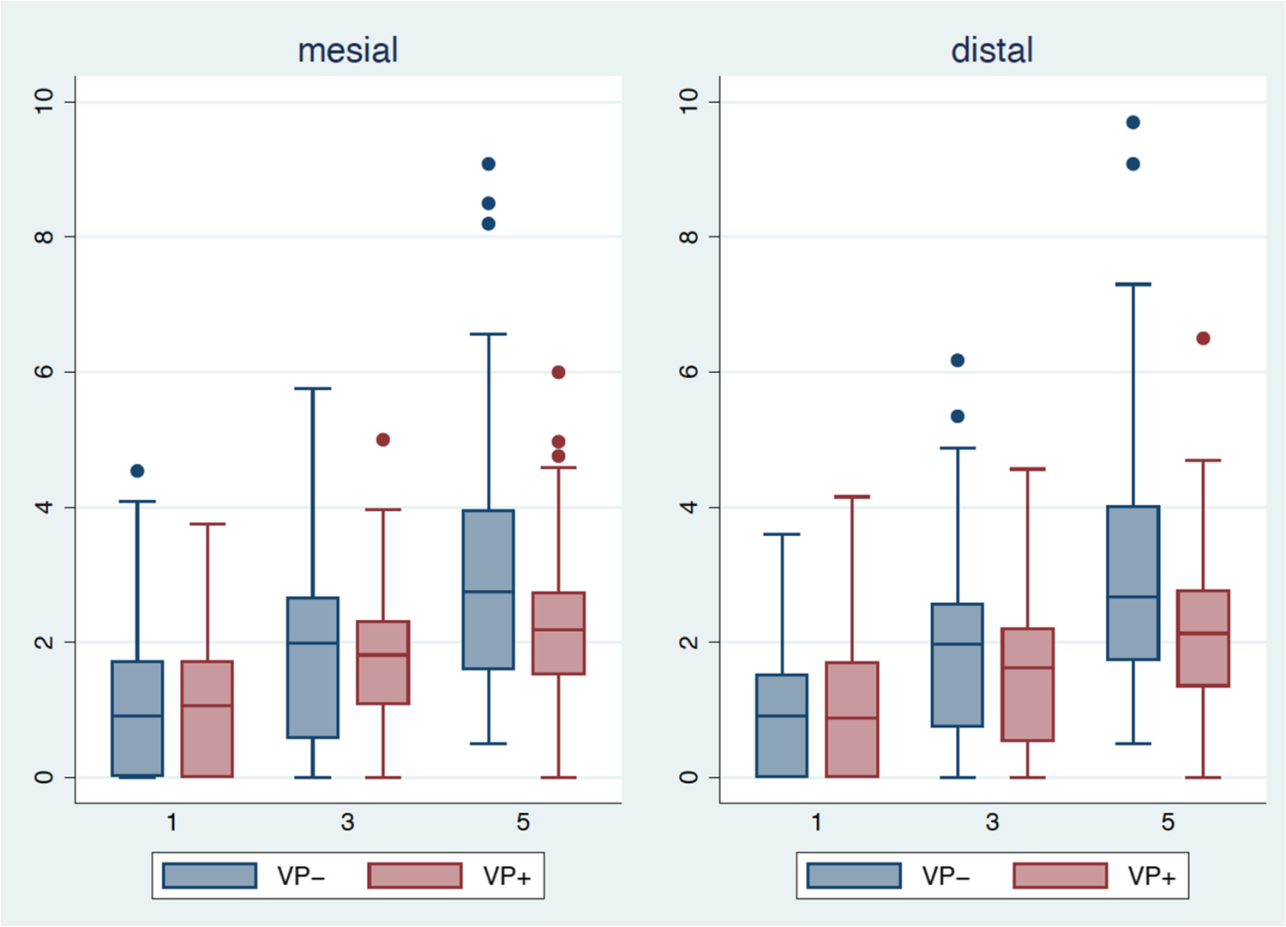
Boxplots representing the bone loss. The mesial (left) and distal (right) peri-implant bone loss after 1, 3, and 5 years in patients with (red, VP +) or without (blue, VP-) vestibuloplasty

**Fig. 4:**
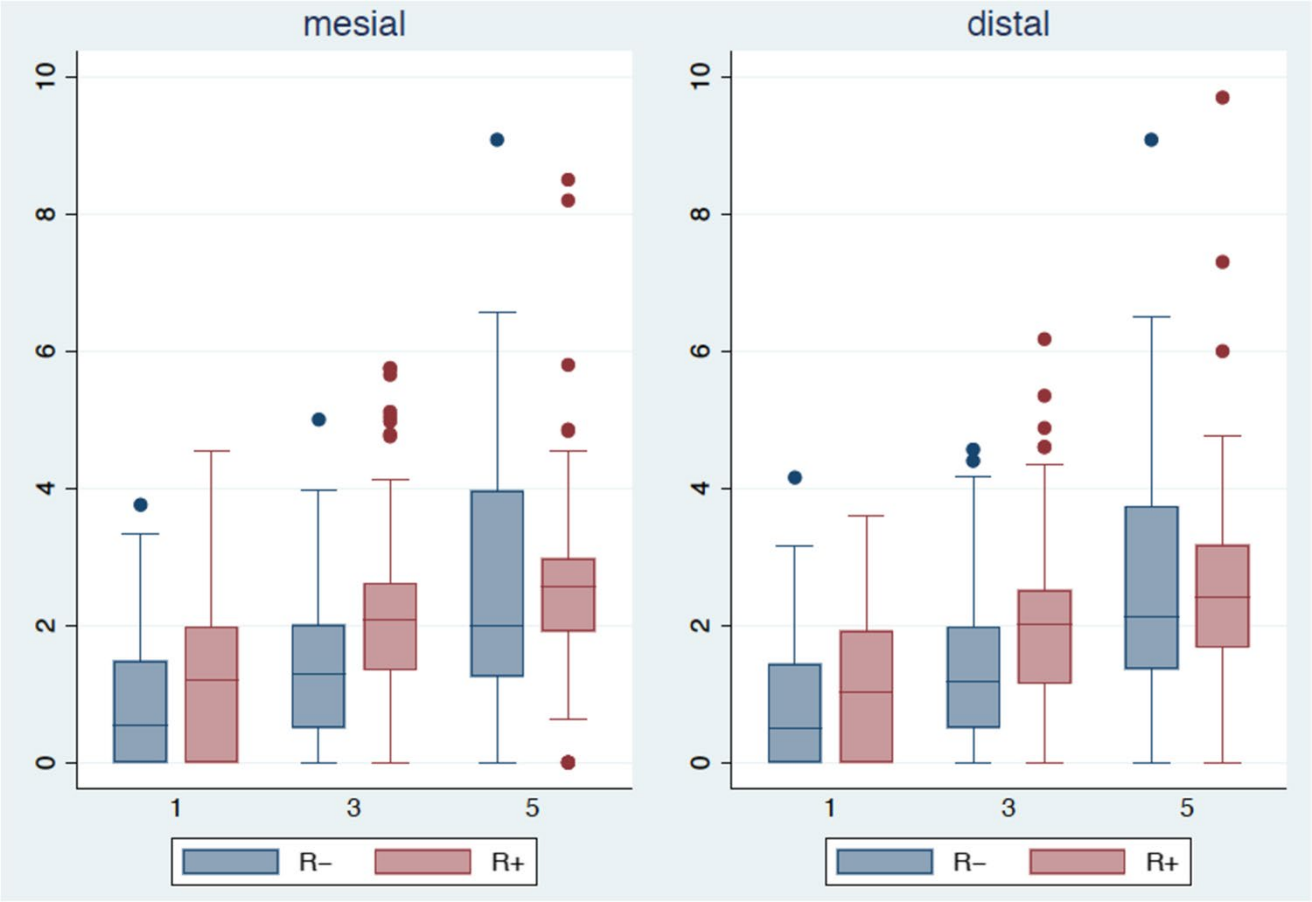
Mesial (left) and distal (right) peri-implant bone loss after 1, 3, and 5 years in irradiated (red, R +) or non-irradiated patients (blue, R-)

Univariate statistical analysis showed significantly higher mesial and distal bone loss in the maxilla compared to the mandible (mesial: *p* = *0.04*; distal: *p* = *0.003*). In patients with VP, (distal) bone loss was found to be significantly lower (*p* = *0.001*) compared to that in patients without VP. Subsequent multivariate analysis showed the influence of VP on bone loss after 5 years, with significantly lower mesial and distal bone loss in patients with VP (mesial: *p* = *0.003*; distal: *p* = *0.001*). The differences in bone loss over time in patients with and without VP appeared to be statistically significant (mesial: *p* = *0.0008*; distal *p* < *0.0001*). Radiation caused a significant change in mesial bone loss after 3 years (*p* = *0.04)*. Peri-implant bone loss was also compared between implants placed in native bone (214 dental implants) and FFF bone flaps (33 dental implants) after 1, 3, and 5 years. The groups showed no significant differences after 1 year, but a significantly lower bone loss after 5 years (mesial: *p* = *0.05*; distal: *p* = *0.44*) was observed in implants inserted in the FFF. Instead of: The groups showed no significant differences after 1 year, but a significantly lower bone loss after 3 years (mesial and distal; *p* = *0.047*) and 5 years (mesial: *p* = *0.05*; distal: *p* = *0.44*) was observed in implants inserted in the FFF. The results are presented in Table 1, Figs. 3 and 4.

**Table 1:** Mesial and distal peri-implant bone loss after 1, 3, and 5 years

	1 year				3 years				5 years			
	Mesial		Distal		Mesial		Distal		Mesial		Distal	
	Mean	± SD	Mean	± SD	Mean	± SD	Mean	± SD	Mean	± SD	Mean	± SD
**Overall**	1.1	1.0	1.0	1.0	1.8	1.3	1.7	1.2	2.6	1.6	2.5	1.7
**R-**	0.9	0.9	0.8	0.9	1.3	1.1	1.3	1.1	2.6	2.0	2.6	1.9
**R + **	1.2	1.1	1.1	1.0	2.1	1.3	2.0	1.3	2.6	1.4	2.5	1.5
**VP-**	1.1	1.1	1.0	0.9	1.9	1.5	1.9	1.4	3.1	2.0	3.1	1.9
**VP + **	1.0	1.0	1.0	1.0	1.7	1.0	1.5	1.1	2.2	1.4	2.1	1.3
**VP- R + **	1.5	0.7	1.3	1.0	2.5	1.5	2.5	1.5	3.1	1.7	3.1	1.8
**VP + R + **	1.0	1.0	1.0	0.9	1.7	0.9	1.7	0.9	2.2	1.0	2.1	1.1
**NB**	1.1	1.0	1.0	1.0	1.7	1.2	1.7	1.1	2.6	1.6	2.6	1.7
**FB**	1.2	1.0	1.1	0.9	2.3	2.0	2.1	1.9	2.3	1.8	2.3	1.7

*R-* non-irradiated patients,* R* +  irradiated patients,* VP-* patients without vestibuloplasty,* VP* +  patients with vestibuloplasty,* VP*- *R* +  irradiated patients without vestibuloplasty,* VP* + *and R* +  irradiated patients with vestibuloplasty, *NB* native bone,* FB* fibula bone

Clinical parameters such as mPi, mBi, PD, and AG were measured after 5 years. mPi had a mean of 0.63 ± 0.74 for all patients, 0.73 ± 0.73 for patients without VP, and 0.54 ± 0.76 for patients with VP. Overall, mBi demonstrated a mean of 0.49 for all patients, 0.54 for patients without VP, and 0.43 for patients with VP. Mean PD was 2.13 mm for all patients, 2.35 mm for patients without VP, and 1.99 mm for patients with VP. Attached keratinized gingiva had a mean of 2.16 mm in all patients, 1.5 mm in patients without VP, and 2.61 mm in patients with VP.

## Discussion

The peri-implant soft tissue and hard tissue is often neglected in head and neck tumor patients despite its well-known importance and positive effects on implant survival and success [2]. Therefore, this study aimed to provide a more comprehensive picture of the peri-implant hard and soft tissue in patients after tumor therapy in the head and neck region, with special attention given to the long-term influence of VP.

Factors such as gender have not been found to affect implant survival in long-term studies﻿ ﻿[22, 23], which is in line with our findings. However, results for patients with additional radiation/radiochemotherapy are inconsistent [22–24]. To date, most studies distinguish between dental implant success and dental implant survival [25] with a focus on one or the other [22, 24, 26–28]. The present study reveals a 100% rate of both cumulative survival and cumulative success after 5 years for dental implants in patients who received VP, with a mean observation period of 58.5 months (follow-up continued for up to 105 months). These results show a high survival rate for implants, unlike numerous other studies with low implant survival rates and comparable follow-up periods [22, 29–31]. Laverty et al. demonstrated a 95.5% long-term survival of dental implants after 5 years [32], which is in line with our findings concerning patients without VP (survival rate of 93.1% after 5 years). Our study revealed a significantly higher implant survival rate for patients with VP. Additionally, patients with VP showed significantly lower mesial and distal peri-implant bone loss during the observation period, more stable clinical parameters (mPi, MBI, and PD), and a greater width of AG around dental implants than patients without VP. Previous studies [10, 33, 34] have highlighted the importance of keratinized gingiva in peri-implant tissue health [35] and peri-implant bone level. The importance of healthy peri-implant soft tissue and stable bone levels in yielding reliable implant success/survival rates is evident in patients with complicated anatomical conditions who have received tumor therapy. The statistically significantly lower peri-implant bone loss after 5 years in VP patients implies the crucial advantage offered by VP in long-term implant survival success.

Since primary or secondary reconstruction of the jaw is often required after tumor resection [36], the clinical parameters of a dental implant inserted in autologous bone flaps is another point of interest. FFF remains the most used bony flap for the reconstruction of the jaw, as it offers sufficient pedicle length and the option of being segmented to follow the shape of the mandible [25, 37–39]. Furthermore, dental implants inserted in the fibula bone have already been proven to be reliable for dental rehabilitation in the long term [40, 41]. For selected cases of reconstructed mandibles with FFF, Kumar et al. introduced an easy and predictable method of achieving healthy peri-implant tissues. The described technique contains buccal and lingual flaps, and an implant placement is done while an interim denture is immediately loaded onto the implants. This enables a guided epithelium regeneration in the area of the FFF [9]. Moreover, proteome analysis revealed that de novo regenerated mucosa over the FFF adopts active tissue function and resembles oral keratinized mucosa [42]. This could create additional possibilities in the future for dental implant treatment in patients after FFF. Since these studies only refer to patients with FFF and data on the resultant effect on dental implant survival and success is still missing, further research is highly welcome.

Previous studies examining peri-implant bone loss in microvascular FFF demonstrated bone-level changes of 0.68–0.71 mm after 3 years [43] and, for non-vascularized fibula bone flaps used in edentulous patients, a mean bone resorption after 10 years of 1.4 mm both in the mesial and the distal [44]. Li et al. examined the marginal bone loss of dental implants in patients who received vascularized-free flaps and free gingival grafts or apical reposition flaps. A marginal bone loss of 0.6 ± 0.4 mm and a probing depth of 3.5 ± 0.9 mm occurred, which can be explained by the shorter observation period, a lack of information on the insertion depth, and the limited number of cancer patients included (15.8%) [45]. Oral hygiene is often difficult to maintain in tumor patients postoperatively [46]; moreover, in cases of irradiation, the peri-implant bone level decreases, according to the specific radiation dose in the peri-implant area [47, 48]. This might explain the constantly lower peri-implant bone loss characterized by inter-individual fluctuations.

Unlike comparable long-term studies that found significantly lower implant survival rates in irradiated jaws [23, 49], our study found no significant differences between implant survival and implant success in irradiated and non-irradiated patients. The results of this study may be explained by the effective radiation dosage, which is known to influence osseointegration and healing after dental implantation [26, 48, 50]. Additionally, irradiated patients were only included if they could be characterized as non-smokers, while non-irradiated patients were only included if they belonged to the category of “very light daily smokers” [15]. Javed et al. posited that radiation dosages between 50 and 65 Gy do not negatively influence osseointegration [26], which could explain our findings. While numerous studies have focused on the influence of radiation on dental implant survival, only a few published studies on chemotherapy and dental implants—most of which are characterized by a low level of specificity and a limited number of cases, without a control group [51]—exist. Comparing our findings (i.e., an implant survival rate of 100% in non-irradiated patients versus 95.7% in irradiated patients after 5 years) to these studies, it appears that the lower survival rate in irradiated patients is mainly an unintended effect of radiation rather than a consequence of the adjunctive chemotherapy.

The results of our study indicate the major advantage of VP as a pre-prosthetic procedure for patients after surgical ablative therapy and (in most cases) radiochemotherapy. Therefore, to achieve satisfactory and—most importantly—stable peri-implant soft tissue and bone conditions in our patient populations, VP should always be considered before prosthetic rehabilitation. This is especially true when patients suffer from altered intraoral anatomy and a deepened vestibulum that features a high-quality level of peri-implant keratinized gingiva, and a stable denture-bearing area is required during the pre-prosthetic stage.

## Conclusion

Dental implant success and survival rates were found to be significantly higher in patients with VP. Additionally, mesial and distal peri-implant bone loss were significantly lower in patients with VP after 5 years. Therefore, VP should always be considered and applied if required by the anatomical situations of individual patients, in order to achieve high implant survival/success rates in head and neck tumor patients. Exclusion of irradiated patients who smoke, patient education, and a regular recall system seem to be indispensable.

## Data Availability

is upon request to the corresponding author.

## References

[1] Lorenz J, Blume M, Barbeck M, Teiler A, Kirkpatrick CJ, Sader RA, Ghanaati S (2017). Expansion of the peri-implant attached gingiva with a three-dimensional collagen matrix in head and neck cancer patients-results from a prospective clinical and histological study. Clin Oral Invest.

[2] Algraffee H, Borumandi F, Cascarini L (2012). Peri-implantitis. Br J Oral Maxillofac Surg.

[3] Galarraga-Vinueza ME, Tavelli L (2022). Soft tissue features of peri-implant diseases and related treatment. Clin Implant Dent Relat Res.

[4] Artzi Z, Tal H, Moses O, Kozlovsky A (1993). Mucosal considerations for osseointegrated implants. J Prosthet Dent.

[5] Heberer S, Nelson K (2009). Clinical evaluation of a modified method of vestibuloplasty using an implant-retained splint. J Oral Maxillofac Surg.

[6] Bassetti RG, Stähli A, Bassetti MA, Sculean A (2017). Soft tissue augmentation around osseointegrated and uncovered dental implants: a systematic review. Clin Oral Invest.

[7] Reddy VK, Parthasarathy H, Lochana P (2013). Evaluating the clinical and esthetic outcome of apically positioned flap technique in augmentation of keratinized gingiva around dental implants. Contemp Clin Dent.

[8] Herford AS, Tandon R, Pivetti L, Cicciù M (2015). Pedicled lingual flap to provide keratinized tissue regeneration over dental implants: a description of the technique and a case report. J Oral Implantol.

[9] Kumar VV, Jacob PC, Kuriakose MA (2016). Sub-periosteal dissection with denture-guided epithelial regeneration: a novel method for peri-implant soft tissue management in reconstructed mandibles. J Maxillofac Oral Surg.

[10] Thoma DS, Naenni N, Figuero E, Hämmerle CHF, Schwarz F, Jung RE, Sanz-Sánchez I (2018). Effects of soft tissue augmentation procedures on peri-implant health or disease: a systematic review and meta-analysis. Clin Oral Implants Res.

[11] Schmitt CM, Moest T, Lutz R, Wehrhan F, Neukam FW, Schlegel KA (2016). Long-term outcomes after vestibuloplasty with a porcine collagen matrix (Mucograft(®) ) versus the free gingival graft: a comparative prospective clinical trial. Clin Oral Implants Res.

[12] Metin M, Dolanmaz D, Alkan A (2003). Evaluation of autogenous grafts used in vestibuloplasty. J Int Med Res.

[13] Shan X, Han D, Ge Y, Zhang H, Lu R (2022). Clinical outcomes of keratinized mucosa augmentation in jaws reconstructed with fibula or iliac bone flaps. Int J Oral Maxillofac Surg.

[14] Kao SY, Lui MT, Fong J, Wu DC, Wu CH, Tu HF, Hung KF, Yeung TC (2005). A method using vestibulo-sulcoplasty combining a split-thickness skin graft and a palatal keratinized mucosa graft for peri-implant tissue secondary to oral cancer surgery. J Oral Implantol.

[15] Ashe ML, Wilson SJ (2021). Very light daily smoking in young adults: relationships between nicotine dependence and lapse. Nicotine Tob Res.

[16] Nack C, Raguse J-D, Stricker A, Nelson K, Nahles S (2015). Rehabilitation of irradiated patients with chemically modified and conventional SLA implants: five-year follow-up. J Oral Rehabil.

[17] Mombelli A, van Oosten MA, Schurch E, Land NP (1987). The microbiota associated with successful or failing osseointegrated titanium implants. Oral Microbiol Immunol.

[18] Peters, Siegwart (1978) Prophylaxe: ein Leitfaden für die zahnärztliche Praxis. Buch-und Zeitschriften-Verlag Die Quintessenz, Berlin, Chicago, Rio de Janeiro, Tokio

[19] Gomez-Roman G, Axmann D, d’Hoedt B, Schulte W (1995). Eine Methode zur quantitativen Erfassung und statistischen Auswertung des periimplantären Knochenabbaues. Stomatologie.

[20] Ernst N, Sachse C, Raguse JD, Stromberger C, Nelson K, Nahles S (2016). Changes in peri-implant bone level and effect of potential influential factors on dental implants in irradiated and nonirradiated patients following multimodal therapy due to head and neck cancer: a retrospective study. J Oral Maxillofac Surg.

[21] Buser D, Ingimarsson S, Dula K, Lussi A, Hirt HP, Belser UC (2002). Long-term stability of osseointegrated implants in augmented bone: a 5-year prospective study in partially edentulous patients. Int J Periodontics Restorative Dent.

[22] Yerit KC, Posch M, Seemann M, Hainich S, Dörtbudak O, Turhani D, Ozyuvaci H, Watzinger F, Ewers R (2006). Implant survival in mandibles of irradiated oral cancer patients. Clin Oral Implants Res.

[23] Doll C, Nack C, Raguse J-D, Stricker A, Duttenhoefer F, Nelson K, Nahles S (2015). Survival analysis of dental implants and implant-retained prostheses in oral cancer patients up to 20 years. Clin Oral Invest.

[24] Linsen SS, Martini M, Stark H (2012). Long-term results of endosteal implants following radical oral cancer surgery with and without adjuvant radiation therapy. Clin Implant Dent Relat Res.

[25] Pellegrino G, Tarsitano A, Ferri A, Corinaldesi G, Bianchi A, Marchetti C (2018). Long-term results of osseointegrated implant-based dental rehabilitation in oncology patients reconstructed with a fibula free flap. Clin Implant Dent Relat Res.

[26] Javed F, Al-Hezaimi K, Al-Rasheed A, Almas K, Romanos GE (2010). Implant survival rate after oral cancer therapy: a review. Oral Oncol.

[27] Schiegnitz E, Al-Nawas B, Kämmerer PW, Grötz KA (2014). Oral rehabilitation with dental implants in irradiated patients: a meta-analysis on implant survival. Clin Oral Invest.

[28] Laverty DP, Addison O, Wubie BA, Heo G, Parmar S, Martin T, Praveen P, Pearson D, Newsum D, Murphy M, Bateman G (2019). Outcomes of implant-based oral rehabilitation in head and neck oncology patients-a retrospective evaluation of a large, single regional service cohort. Int J Implant Dent.

[29] Schliephake H, Neukam FW, Schmelzeisen R, Wichmann M (1999). Long-term results of endosteal implants used for restoration of oral function after oncologic surgery. Int J Oral Maxillofac Surg.

[30] Nelson K, Heberer S, Glatzer C (2007). Survival analysis and clinical evaluation of implant-retained prostheses in oral cancer resection patients over a mean follow-up period of 10 years. J Prosthet Dent.

[31] Curi MM, Condezo AFB, Ribeiro K, Cardoso CL (2018). Long-term success of dental implants in patients with head and neck cancer after radiation therapy. Int J Oral Maxillofac Surg.

[32] Laverty DP, Addison O, Wubie BA, Heo G, Parmar S, Martin T, Praveen P, Pearson D, Newsum D, Murphy M, Bateman G (2019). Outcomes of implant-based oral rehabilitation in head and neck oncology patients—a retrospective evaluation of a large, single regional service cohort. Int J Implant Dent.

[33] Bouri A, Bissada N, Al-Zahrani MS, Faddoul F, Nouneh I (2008). Width of keratinized gingiva and the health status of the supporting tissues around dental implants. Int J Oral Maxillofac Implants.

[34] Lin G-H, Chan H-L, Wang H-L (2013). The significance of keratinized mucosa on implant health: a systematic review. J Periodontol.

[35] Ladwein C, Schmelzeisen R, Nelson K, Fluegge TV, Fretwurst T (2015). Is the presence of keratinized mucosa associated with periimplant tissue health? A clinical cross-sectional analysis. Int J Implant Dent.

[36] Huang TH, Kuo PJ, Liu CJ (2021). Comparison of surgical outcomes between primary plate and fibular flap transfer for reconstruction of segmental mandibular defects. Microsurgery.

[37] Hidalgo DA, Rekow A (1995). A review of 60 consecutive fibula free flap mandible reconstructions. Plast Reconstr Surg.

[38] Urken ML, Buchbinder D, Costantino PD, Sinha U, Okay D, Lawson W, Biller HF (1998). Oromandibular reconstruction using microvascular composite flaps: report of 210 cases. Arch Otolaryngol Head Neck Surg.

[39] Lonie S, Herle P, Paddle A, Pradhan N, Birch T, Shayan R (2016). Mandibular reconstruction: meta-analysis of iliac- versus fibula-free flaps. ANZ J Surg.

[40] Fang W, Liu YP, Ma Q, Liu BL, Zhao Y (2015). Long-term results of mandibular reconstruction of continuity defects with fibula free flap and implant-borne dental rehabilitation. Int J Oral Maxillofac Implants.

[41] Goker F, Baj A, Bolzoni AR, Maiorana C, Giannì AB, Del Fabbro M (2020). Dental implant-based oral rehabilitation in patients reconstructed with free fibula flaps: clinical study with a follow-up 3 to 6 years. Clin Implant Dent Relat Res.

[42] Kumar VV, James BL, Ruß M, Mikkat S, Suresh A, Kämmerer PW, Glocker MO (2018). Proteome analysis reveals that de novo regenerated mucosa over fibula flap-reconstructed mandibles resembles mature keratinized oral mucosa. Oral Oncol.

[43] Wang F, Huang W, Zhang C, Sun J, Kaigler D, Wu Y (2015). Comparative analysis of dental implant treatment outcomes following mandibular reconstruction with double-barrel fibula bone grafting or vertical distraction osteogenesis fibula: a retrospective study. Clin Oral Implants Res.

[44] Duttenhoefer F, Nack C, Doll C, Raguse JD, Hell B, Stricker A, Nelson K, Nahles S (2015). Long-term peri-implant bone level changes of non-vascularized fibula bone grafted edentulous patients. J Cranio-Maxillo-fac Surg.

[45] Li R, Meng Z, Zhang Y, Shan X, Wang Y, He Y (2021). Soft tissue management: a critical part of implant rehabilitation after vascularized free-flap reconstruction. J Oral Maxillofac Surg.

[46] Matsuda Y, Okui T, Karino M, Aoi N, Okuma S, Hayashida K, Sakamoto T, Kanno T (2021). Postoperative oral dysfunction following oral cancer resection and reconstruction: a preliminary cross-sectional study. Oral Oncol.

[47] Neckel N, Wagendorf P, Sachse C, Stromberger C, Vach K, Heiland M, Nahles S (2021). Influence of implant-specific radiation doses on peri-implant hard and soft tissue: an observational pilot study. Clin Oral Implants Res.

[48] Sammartino G, Marenzi G, Cioffi I, Teté S, Mortellaro C (2011). Implant therapy in irradiated patients. J Craniofac Surg.

[49] Gupta S, Mortellaro C, Panda S et al (2021) Dental implant survival rate in irradiated and non-radiated patients: a systematic review and meta-analysis. J Biol Regul Homeost Agents 35(2 Suppl. 1):53–65. 10.23812/21-2supp1-5 10.23812/21-2supp1-5 34281302

[50] Granström G, Jacobsson M, Tjellström A (1992). Titanium implants in irradiated tissue: benefits from hyperbaric oxygen. Int J Oral Maxillofac Implants.

[51] Chrcanovic BR, Albrektsson T, Wennerberg A (2016). Dental implants in patients receiving chemotherapy: a meta-analysis. Implant Dent.

